# The Relationship of Physical Activity and Dietary Quality with Android Fat Composition and Distribution in US Adults

**DOI:** 10.3390/nu14142804

**Published:** 2022-07-08

**Authors:** Furong Xu, Jacob E. Earp, Dara L. LoBuono, Geoffrey W. Greene

**Affiliations:** 1 School of Education, University of Rhode Island, Kingston, RI 02881, USA; 2 Department of Kinesiology, University of Connecticut, Storrs, CT 06269, USA; jacob.earp@uconn.edu; 3 Department of Health and Exercise Science, Rowan University, Glassboro, NJ 08028, USA; lobuono@rowan.edu; 4 Department of Nutrition and Food Sciences, University of Rhode Island, Kingston, RI 02881, USA; ggreene@uri.edu

**Keywords:** android fat, android/gynoid ratio, physical activity, dietary quality, adults

## Abstract

This study examined the relationship of physical activity (PA) and dietary quality to android fat composition and distribution using a national representative adult sample and determined sex-based differences in these relationships. It is a cross-sectional (*n* = 10,014) analysis of the 2011–2018 National Health and Nutrition Examination Survey and the US department of Agriculture’s Food Patterns Equivalents datasets. Variables utilized for this analysis include PA, 24-h dietary recalls, android percent fat, and android-gynoid (A/G) ratio measured by dual-energy X-ray absorptiometry. Multiple linear regression was performed to examine the relationship between PA and/or dietary quality and android percent fat and A/G ratio adjusted for confounding factors. The study results revealed that PA and/or dietary quality were inversely related to android percent fat and A/G ratio (*p* < 0.05), but the sex effect was only seen between PA and A/G ratio (*p* = 0.003). Participants who met PA recommendations and had higher dietary quality had 2.12% lower android fat than those who did not meet PA recommendations and had lower dietary quality (*p* < 0.001). Both PA and dietary quality are associated with android fat reduction regardless of sex. Given the direct connection between android fat and cardiovascular and metabolic diseases, it is important to increase both PA and dietary quality.

## 1. Introduction

The rapidly increasing rate of obesity is anticipated to have serious negative health consequences [1,2]. Research has indicated that excess body fat is associated with many metabolic and cardiovascular risk factors [3]. However, more recent research indicated that fat centralized in the abdominal/android region is a more sensitive indicator of metabolic or cardiovascular risk for chronic diseases than overall body fat percentage or fat located in the hip/gynoid region [4,5,6,7,8]. Specifically, studies have found that android accumulation was associated with metabolic risk factors [4,5], was a better estimator for the risk of type II diabetes than overall fat mass [6,7,8] and was a risk factor for stroke independent of body mass index [9]. It is important to understand how health behaviors influence android fat composition and its ratio to gynoid fat mass (A/G ratio), thus indirectly affecting related health risks.

Previous studies have found that higher levels of physical activity are related to reduced abdominal fat and A/G ratio [10,11,12,13,14]. Previous studies also found dietary quality, which refers to the balanced and diversified dietary patterns, used to evaluate compliance to dietary guidelines [15], is related to abdominal fat and A/G ratio [16,17,18,19,20]. While larger scale studies investigating the influence of health behaviors on A/G ratio are lacking, two studies examined such relationships with consideration of physical activity intensity [11,18]. More specifically, one study reported moderate to vigorous physical activity is inversely associated with A/G ratio in adults, 60–64 years, in mainland Britain, [11] while another study reported inverse relationships between dietary quality and A/G ratio in 60 years of age or older Australian males [18]. However, to our knowledge, neither of these previous studies examined the relationship between physical activity or dietary quality and android fat or the A/G ratio using a representative sample of US young and middle-aged adults. In addition, studies are also lacking in examining dose-response relationship between physical activity or dietary quality and android fat composition and distribution in this population. This is an important research gap to address as young and middle-aged adults are also age groups facing obesity related health concerns as they age [21,22,23]. Lifestyle habits developed during this age period can be maintained in older age [11,24], thus can help better reduce health challenges in an aging population. Furthermore, it is presently unknown if sex influences the relationships between health behaviors and fat distribution (A/G ratio). A better knowledge base of sex-based differences can help practitioners and policy makers prioritize lifestyle modifications and guide investment in public health campaigns with the greatest epidemiological benefit. Accordingly, more studies are needed on the relationship of physical activity and/or dietary quality with android fat and the A/G ratio using a representative sample of US adults.

## 2. Methods

**Figure 1:**
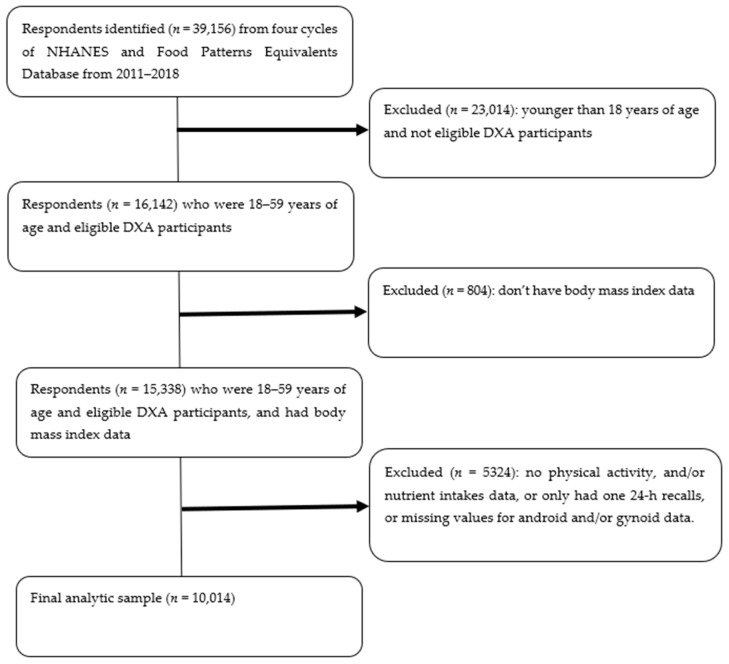
Study flow chart. *Note:* NHANES = National Health and Nutrition Examination Survey, DXA = dual-energy X-ray absorptiometry.

The current study is a secondary data analysis utilizing four cycles and eight years of data (2011–2018) from two datasets: (1) National Health and Nutrition Examination Survey (NHANES) and (2) the US Department of Agriculture’s Food Patterns Equivalents [25,26]. The inclusion criteria for this study are adults who have (1) dual-energy X-ray absorptiometry (DXA) data (18–59 years old; *n* = 16,142), and data for (2) body mass index (*n* = 15,338), (3) physical activity, two 24-h dietary recalls (*n* = 11,374), and android and gynoid fat mass (*n* = 10,014). Accordingly, 10,014 out of 39,156 respondents met all the above inclusion criteria and have been included for the current study (see Figure 1). The University of Rhode Island Institutional Review Board has approved the current study under exemption category (IRB reference#: 1849196-1).

### 2.1. Android Fat Composition and Distribution

Android and gynoid fat were measured by using DXA and analyzed via the Hologic APEX software [25,27]. In the Hologic software, both android and gynoid regions were defined in the same method as utilized in Shepherd and colleagues’ study [28]. The android area was defined as the lower trunk area above the pelvic line and 20% of the distance between this line and the neck cut line, and gynoid area was defined as twice the height of the android region below the pelvic line [28]. Android percent fat was defined as android fat mass divided by android total mass; and A/G ratio was calculated by the Hologic APEX software used in the scan analysis [25].

### 2.2. Physical Activity

The Global Physical Activity questionnaire was used to measure a typical week of physical activity which was analyzed following the World Health Organization analysis guide for Global Physical Activity questionnaire [25,29]. Physical activity results are reported as metabolic equivalent (MET) minutes of moderate to vigorous physical activity per week. MET-minutes per week was used as opposed to physical activity time (min/week) or energy expenditure (kcal/week) as this variable accounts for the variation in metabolic demands between a wide range of common physical activities that is independent of body mass of the individual [29]. Results were classified as three categories: insufficiently active (<600 MET-minutes/week), active (600–1200 MET-minutes/week) and highly active (>1200 MET-minutes/week) based on U.S. Department of Health and Human Services’ physical activity guidelines for Americans [30]. Respondents classified as active and highly active met the current physical activity recommendation of 600 MET-minutes/week for adults [30].

### 2.3. Dietary Quality

Dietary quality was measured using the Healthy Eating Index 2015 (HEI-2015) which is based on adherence to the 2015–2020 Dietary Guidelines for Americans [31]. Assessing adherence to the dietary guidelines provides a broad picture of dietary patterns and consumption, which is more predictive of disease risks than focusing on consumption of individual nutrients [32]. The HEI-2015 analysis used data from two datasets: National Health and Nutrition Examination Survey’s two 24-h dietary recalls and US Department of Agriculture’s Food Patterns Equivalents dataset [25,26]. The HEI-2015 scoring metric contains 13 dietary components that assess either adequacy (9 components) or moderation of intake (4 components) [31]. The maximum score for HEI-2015 is 100 which has been classified into three categories for this study based on the score distribution in this sample: Higher dietary quality (the highest tertile, 58.1 < HEI-2015 ≤ 95.8), Lower dietary quality (the lower two tertiles, 10 ≤ HEI-2015 ≤ 58.1). This approach has been used previously to analyze HEI-2015 data [33].

### 2.4. Lifestyle Groups

Four lifestyle groups were categorized utilizing the criteria for physical activity recommendation (met vs. did not meet) and dietary quality score distribution (higher vs. lower) [34]. More specifically, (1) Group 1: did not meet physical activity recommendation + lower dietary quality, (2) Group 2: did not meet physical activity recommendation + higher dietary quality, (3) Group 3: met physical activity recommendation + lower dietary quality, (4) Group 4: met physical activity recommendation + higher dietary quality.

### 2.5. Confounding Variables

Respondents’ demographics characteristics were reported for the current study are age, race/ethnicity (White, Black, Hispanic, others), education (high school or less, some college or more), and ratio of family income to poverty [25]. Body mass index was also reported and has been further classified as underweight, normal, overweight, and obese based on Centers for Disease Control and Prevention’s body mass index interpretation for adults [35]. Additionally, daily energy intake has also been included due to its possible influence on our study variables [36].

### 2.6. Data Analysis

All analyses were performed using the combined 8-year sample weights. The dietary two-day sample weight was selected to construct weights for the combination of four data cycles (2 years per cycle & 8 years in total) based on National Health and Nutrition Examination Survey Methods and Analytic Guidelines regarding weight selection and weight construction for combined data cycles [37]. Multicollinearity among independent variables (physical activity or HEI score) and control covariates were checked using PROC REG with weight statement, no collinearities were observed using the criteria based on the condition index exceeding 30 [38]. Sample characteristics are expressed as weighted means ± standard errors or count (percentage). *p* values for continuous variables were obtained by performing *t*-test (PROC SURVEYREG), and *p* values for categorical variables were obtained by performing Chi-Squared test (PROC SURVEYREQ). For the relationships between physical activity and/or dietary quality and android fat composition and distribution, adjusted β (95% confidence interval), *p* values and R-square were obtained performing multiple linear regression (PROC SURVEYREG) adjusted for age, sex, race/ethnicity, education, family income to poverty ratio, body mass index and daily energy intake. The interaction terms were added into the model to examine the modification effect of sex to investigate whether the association between physical activity and/or dietary quality and android fat composition and distribution differed by male and female. Statistical Analysis Software 9.4 (SAS Institute Inc., Cary, NC, USA) was utilized to analyze the data considering the complex sample design, and *p* < 0.05 was chosen as the statistically significant level.

## 3. Results

Out of 10,014 respondents, approximately half (48.7%) of them are females, 39.3% are racial/ethnic minorities; 32.9% have high school or less education, 15.9% live below poverty level; 1.6%, 31.5% and 37.3% are respondents whose body mass index categorized as underweight, overweight and obese respectively. Additionally, 31% do not meet physical activity recommendation, and by definition 66.0% had HEI scores in the lower dietary quality category. Males have lower android fat percentage and are more physically active than females whereas females have a lower A/G ratio and better dietary quality than males (see Table 1). Moreover, the descriptive results in Table 2 indicated that there was a linear decrease in android fat percentage from lower to higher physical activity and dietary quality groups for both sexes with the lowest android fat percentage seen in the met physical activity recommendations and higher dietary quality group (see Table 2).

There was an inverse relationship between physical activity and android fat composition and distribution (see Table 3 and Table 4). For every 100 MET-minutes/week increase, the percent android fat was reduced by 0.0103. Respondents who were classified as highly active had 1.64 percent lower android fat on average than those who were classified as insufficiently active. No statistically significant difference was observed between physical activity and the A/G ratio (see Table 3). There was a similar pattern observed in males and females; however, there were differences in A/G ratio as there was a significant interaction by sex (see Table 4). For every 100 MET-minutes/week physical activity increase, males had a greater A/G ratio reduction (β = −0.0002, 95% CI: −0.0004, −0.0001) than females (see Table 4).

Similar to physical activity, there was an inverse relationship between dietary quality and android fat composition and distribution (see Table 3). For every 10-point HEI score increase, android fat decreased by 0.34%, and the A/G ratio decreased 0.01 on average. In comparison to those with a lower dietary quality score, respondents who had higher dietary quality scores showed lower android percent fat. No statistically significant differences in the A/G ratio were observed between higher and lower dietary quality score groups (see Table 3). However, the results in sex specific analyses revealed that females with higher HEI scores showed lower A/G ratio compared to those who had lower HEI scores but there was no sex by score group interaction (see Table 4).

Physical activity and diet integrated lifestyle group analyses indicated that in comparison to respondents who were in group 1: did not meet physical activity recommendation and had lower dietary quality, those in other lifestyle groups had a lower percentage of android fat. The difference was greatest (2.12% for android fat) between group 1 (not meet physical activity recommendation and lower dietary quality) and group 4 (met physical activity recommendation and had higher dietary quality). There was no difference in A/G ratio between lifestyle groups (see Table 3). There was a similar pattern observed in analysis specifically for males. For females, there was A/G ratio difference between group 4 and group 1. Additionally, compared to their counterparts in group 1, males who met physical activity recommendations but had a lower dietary quality had lower android fat percentage than females (β = −0.86, 95% CI: −1.58, −0.15) (see Table 4).

## 4. Discussion

The key finding of the present study was that both physical activity and dietary quality were inversely related to android percent fat in men and women. Meeting physical activity recommendations and having higher dietary quality provided a compounding impact on reducing android fat percentage. This finding supports multi-component lifestyle modifications that focus on both lifetime physical activity and overall dietary quality in order to reduce android fat and reduce the risk of obesity related chronic diseases. To the authors’ knowledge, this is the first large scale study utilizing a nationally representative sample measuring fat using DXA, leisure time physical activity using a validated measure and dietary quality using the HEI-2015 based on two 24-h recalls. In addition, the study sample of 18–59-year-old adults, is the appropriate target population for primary prevention programs.

In the present study, leisure time physical activity was inversely associated with android percent fat for both sexes. Specifically, respondents showed 0.0103 percent android fat reduction for every 100 MET-minutes/week physical activity time increase which is approximately 123 kcal/week expended for a person who weighs 70 kg, this is equivalent to 25 min/week of moderate intensity (e.g., walking) or 12.5 min/week of vigorous intensity (e.g., running) physical activity [39]. This finding has been supported by previous studies [10,11,12] but adds to the literature with a large representative sample of general adults in comparison to previous studies either using smaller sample sizes [10,11], or special populations such as ethnic Greenlanders [12] or older adults in Britain [11]. The current study extends previous studies by examining a dose-response relationship between android percent fat and physical activity levels (insufficiently active, active, highly active) as defined by U.S. Department of Health and Human Services’ physical activity guidelines for Americans [30]. While the beneficial effects of physical activity on android fat were observed similarly between sexes, people who were categorized as highly active (>1200 MET-minutes/week) have lower android fat than those who were insufficiently active. Moreover, we found that an increase in physical activity time provided greater reductions in the A/G ratio in men than in women. However, it should also be noted that categorical comparisons between sexes revealed that among participants who met physical activity recommendations but had lower dietary quality, men had lower android fat percentage than women, suggesting the effect of physical activity may be stronger in men than women. Nevertheless, these results suggest that both men and women will likely benefit from having an active lifestyle. We believe, these results relate to the importance of physical activity promotion for public health given one third of respondents in the present study reported being insufficiently active.

Similar to physical activity, there was an inverse relationship between dietary quality and android fat composition and distribution. For every 10-point HEI-2015 score increase, android fat decreased by 0.34% and A/G ratio decreased by 0.01. Since the HEI is intended to evaluate consumption of a set of foods in relation to the dietary guidelines, rather than dietary quantity, scores can be interpreted using a graded approach to qualitatively describe adherence to the 2015–2020 Dietary Guidelines for Americans (A = 90–100, B = 80–89, C = 70–79, D = 60–69, and F = 0–59) [40]. Therefore, a 10-point increase can be viewed as an increase in a letter grade and thus steps towards adhering to the dietary guidelines, with scores >80, indicative of close adherence. Typically, individuals can improve their scores by increasing foods from food sources in the adequacy dietary component categories while simultaneously decreasing food sources in the moderation component categories [40]. Regardless, this finding is in line with observations from previous studies [16,18]. Direct comparisons are not possible due to the sample differences (adults vs. older adults) [18] or national vs. regional samples [16] or different dietary quality measures (HEI-2015 scores vs. Mediterranean-style diet) [16]. The study sample utilized NHANES data collected 2011–2018 thus the use of HEI-2015 reflecting 2015–2020 Dietary Guidelines for Americans is appropriate [31]. NHANES dietary data collection methodology is rigorous, and the dietary database was concurrent with data collection adding strength to study findings [25,26]. This study determined the influence of compliance with dietary guidelines, as measured by HEI-2015 scores, on android fat across a representative sample of young to middle aged Americans. The finding that increased HEI-2015 scores were associated with decreased android fat likely reflects the cumulative effects of consumption of a healthier diet because HEI-2015 scores are dependent upon the consumption of a balanced diet, high in nutrient dense foods (e.g., fruits, vegetables, whole grains, dairy, lean proteins and unsaturated fats) and low in refined grains, sodium, sugar and saturated fats [31]. However, future research is needed to investigate if certain dietary components or total diet are more closely related to android fat.

Another important finding of the present study was that the strength of the healthy lifestyle (physical activity and diet) on android fat composition and distribution. The summative effects of both physical activity and dietary quality provide strong evidence that healthy behaviors are multifaceted and that lifestyle modifications made to improve overall health and curb the progression of obesity related illnesses should reflect both physical activity and diet. This finding provides evidence-based justification for health practitioners to prioritize healthy lifestyle that encompasses both physical activity and dietary behaviors. Additionally, while beneficial effects of physical activity and dietary quality on abdominal fat percentage were seen across sexes, it is noteworthy that the A/G ratio differences between lifestyle groups was only observed in females, but not males. When these relationships were directly compared between sexes, the comparison failed to reach significance. These results revealed the variation of android fat or A/G ratio related to dietary quality and physical activity regardless of weight status or energy consumption since both body mass index and daily energy intake were adjusted for in all analyses. It is possible that adjustment might not fully address overall adiposity. For instance, body mass index may over-represent adiposity in those with high muscle mass with low fat mass and under-represent adiposity of those with low muscle mass and high fat mass. In conclusion, further research is needed to determine if changing physical activity and diet will affect android fat similarly in men and women.

## 5. Limitations and Strength

The strengths of the present study are (1) we examined the association of integrated physical activity and dietary quality with android fat using nationally representative data, (2) we were able to effectively compare these associations between sexes, (3) android fat and A/G ratio were measured by using DXA. The limitations of the present study are: (1) its cross-sectional study nature which did not allow determination of causality; (2) the physical activity instrument and two 24-h dietary recalls might possess certain limitations due to the nature of self-report, although these instruments have been validated and widely used in large studies like ours [29,31]; (3) the average HEI score of 52.67 is slightly lower than the national average, thus findings might not be fully representative and generalizable to the US population; (4) there might be a risk of residual confounding factors even though we adjusted for appropriate confounders including body mass index.

## 6. Conclusions

The present study found that physical activity and dietary quality were inversely associated with percent of android fat regardless of sex. However, males experienced greater benefits to the android fat distribution (A/G ratio) than females through increasing weekly physical activity time. Furthermore, respondents who met physical activity recommendations and had higher dietary quality had lowest percentage of android fat (men and women both) and a lower A/G ratio (women only) than respondents who did not meet physical activity recommendations and had lower dietary quality.

## Figures and Tables

**Table 1:** Respondents’ characteristics stratified by sex, NHANES 2011–2018.

Variables	Total	Male	Female	*p* Value
	*n* = 10,014	*n* = 4962 (51.3%)	*n* = 5052 (48.7%)	
Age, *n* (weighted %)				
18–39 yrs	5326 (52.1)	2730 (54.3)	2596 (49.8)	0.002 *
40–59 yrs	4688 (47.9)	2232 (45.7)	2456 (50.2)	0.002 *
Race/ethnicity, *n* (weighted %)				
White	3552 (60.7)	1786 (60.7)	1766 (60.6)	0.921
Black	2286 (12.0)	1089 (11.5)	1197 (12.6)	0.007 *
Hispanic	2425 (17.4)	1150 (17.7)	1275 (17.1)	0.363
Others	1751 (9.9)	937 (10.1)	814 (9.7)	0.491
Education, *n* (weighted %)				
High school or less	3457 (32.9)	1876 (36.0)	1581 (29.7)	<0.001 *
Some college or more	5758 (67.1)	2687 (64.0)	3071 (70.3)	<0.001 *
Ratio of family income to poverty, *n* (weighted %)				
<1.0	2086 (15.9)	945 (14.4)	1141 (17.5)	<0.001 *
≥1.0	7166 (84.1)	3623 (85.6)	3543 (82.5)	<0.001 *
Body Mass Index (kg/m^2^)	28.91 ± 0.15	28.72 ± 0.17	29.11 ± 0.20	0.079
Weight status, *n* (weighted %)				
Underweight	196 (1.6)	86 (1.1)	110 (2.0)	0.008 *
Normal	2927 (29.1)	1373 (26.2)	1554 (32.1)	<0.001 *
Overweight	3026 (31.5)	1734 (36.0)	1292 (26.7)	<0.001 *
Obese	3812 (37.3)	1743 (36.2)	2069 (38.4)	0.17
Daily energy intake (kcal/d)	2158.07 ± 13.80	2478.55 ± 18.05	1820.78 ± 16.94	<0.001 *
Android percent fat	34.91 ± 0.19	31.58 ± 0.22	38.41 ± 0.28	<0.001 *
Gynoid percent fat	35.38 ± 0.15	28.82 ± 0.14	42.29 ± 0.15	<0.001 *
Android to Gynoid ratio	1.00 ± 0.00	1.09 ± 0.01	0.90 ± 0.00	<0.001 *
Dietary quality (HEI-2015)				
Total dietary quality score	52.67 ± 0.33	51.30 ± 0.35	54.11 ± 0.43	<0.001 *
1st tertile (10 ≤ HEI ≤ 45.8), *n* (weighted %)	3337 (32.8)	1796 (36.2)	1541 (29.3)	<0.001 *
2nd tertile (45.8 < HEI ≤ 58.1), *n* (weighted %)	3338 (33.2)	1670 (33.5)	1668 (32.9)	0.67
3rd tertile (58.1 < HEI ≤ 95.8), *n* (weighted %)	3339 (34.0)	1496 (30.3)	1843 (37.8)	<0.001 *
Physical Activity (MET-minutes/week)				
Total Physical Activity	3777.41 ± 106.59	4917.00 ± 178.13	2578.03 ± 88.64	<0.001 *
Insufficiently active (<600)	3387 (31.0)	1288 (24.7)	2099 (37.6)	<0.001 *
Active (600–1200)	1356 (13.3)	577 (11.0)	779 (15.6)	<0.001 *
Highly active (>1200)	5271 (55.8)	3097 (64.3)	2174 (46.8)	<0.001 *
Met PA recommendation (active +highly active), *n* (weighted %)	6627 (69.0)	3674 (75.3)	2953 (62.4)	<0.001 *
PA + Diet, *n* (weighted %)				
Did not meet PA recommendation + lower dietary quality	2354 (22.0)	917 (17.4)	1437 (26.9)	<0.001 *
Did not meet PA recommendation + higher dietary quality	1033 (9.0)	371 (7.3)	662 (10.7)	<0.001 *
Met PA recommendation + lower dietary quality	4321 (44.0)	2549 (52.3)	1772 (35.3)	<0.001 *
Met PA recommendation + higher dietary quality	2306 (25.0)	1125 (23.0)	1181 (27.1)	0.002 *

*Note:* Results expressed as weighted means ± standard errors or count (percentage); NHANES = National Health and Nutrition Examination Survey; PA = physical activity; HEI = Healthy Eating Index; meeting PA recommendation = 600 MET-minutes physical activity time or more each week; Higher dietary quality = 3rd tertile, 58.1 < HEI ≤ 95.8; lower dietary quality = 1st and 2nd tertiles, 10 ≤ HEI ≤ 58.1. * Symbols indicate statistical significance.

**Table 2:** Android fat composition and distribution by physical activity and/or dietary quality levels, NHANES 2011–2018 (*n* = 10,014).

Variable	Android Percent Fat	Android to Gynoid Ratio
	Weighted Mean ± SE	Weighted Mean ± SE
Total	34.91 ± 0.19	1.00 ± 0.00
Physical activity		
Insufficiently active	37.91 ± 0.28	1.01 ± 0.01
Active	35.48 ± 0.41	0.99 ± 0.01
Highly active	33.11 ± 0.23	1.00 ± 0.01
Dietary quality (HEI-2015)		
1st tertile	35.48 ± 0.25	1.02 ± 0.01
2nd tertile	35.24 ± 0.26	1.01 ± 0.00
3rd tertile	34.03 ± 0.28	0.97 ± 0.01
PA + Diet		
Did not meet PA recommendation + lower dietary quality	38.35 ± 0.30	1.01 ± 0.01
Did not meet PA recommendation + higher dietary quality	36.83 ± 0.58	1.00 ± 0.01
Met PA recommendation + lower dietary quality	33.87 ± 0.22	1.01 ± 0.00
Met PA recommendation + higher dietary quality	33.02 ± 0.29	0.96 ± 0.01
Male	31.58 ± 0.22	1.09 ± 0.01
Physical activity		
Insufficiently active	33.77 ± 0.37	1.12 ± 0.01
Active	32.44 ± 0.60	1.11 ± 0.02
Highly active	30.60 ± 0.26	1.08 ± 0.01
Dietary quality (HEI-2015)		
1st tertile	32.09 ± 0.29	1.09 ± 0.01
2nd tertile	31.44 ± 0.35	1.09 ± 0.01
3rd tertile	31.13 ± 0.41	1.09 ± 0.01
PA + Diet		
Did not meet PA recommendation + lower dietary quality	34.05 ± 0.35	1.11 ± 0.01
Did not meet PA recommendation + higher dietary quality	33.09 ± 0.94	1.13 ± 0.02
Met PA recommendation + lower dietary quality	31.03 ± 0.28	1.08 ± 0.01
Met PA recommendation + higher dietary quality	30.50 ± 0.40	1.08 ± 0.01
Female	38.41 ± 0.28	0.90 ± 0.00
Physical activity		
Insufficiently active	40.77 ± 0.30	0.93 ± 0.01
Active	37.74 ± 0.44	0.90 ± 0.01
Highly active	36.74 ± 0.39	0.88 ± 0.01
Dietary quality (HEI-2015)		
1st tertile	39.89 ± 0.40	0.93 ± 0.01
2nd tertile	39.32 ± 0.38	0.92 ± 0.01
3rd tertile	36.48 ± 0.35	0.87 ± 0.01
PA + Diet		
Did not meet PA recommendation + lower dietary quality	41.27 ± 0.36	0.94 ± 0.01
Did not meet PA recommendation + higher dietary quality	39.51 ± 0.58	0.91 ± 0.01
Met PA recommendation + lower dietary quality	38.30 ± 0.39	0.91 ± 0.01
Met PA recommendation + higher dietary quality	35.28 ± 0.35	0.86 ± 0.01

*Note:* Results expressed as weighted means ± standard errors (SE) or count (percentage); NHANES = National Health and Nutrition Examination Survey; PA = physical activity; HEI = Healthy Eating Index; meeting PA recommendation = 600 MET-minutes physical activity time or more each week; Higher dietary quality = 3rd tertile, 58.1 < HEI ≤ 95.8; lower dietary quality = 1st and 2nd tertiles, 10 ≤ HEI ≤ 58.1.

**Table 3:** The relationship of physical activity and/or dietary quality with android fat composition and distribution, NHANES 2011–2018 (*n* = 10,014).

Variable	Android Percent Fat			Android to Gynoid Ratio		
	Adj. β (95% CI)	*p*-Value	R-Square	Adj. β (95% CI)	*p*-Value	R-Square
PA total-per 100-point increase	−0.0103 (−0.0140, −0.0066)	<0.001 *	0.638	−0.0001 (−0.0002, 0.0000)	0.086	0.498
Insufficiently active	Ref	-	0.652	Ref	-	0.502
Active	−0.38 (−0.85, 0.10)	0.12		0.01 (−0.00, 0.03)	0.06	
Highly active	−1.64 (−2.05, −1.23)	<0.001 *		−0.01 (−0.02, 0.01)	0.349	
HEI total-per 10-point increase	−0.34 (−0.49, −0.19)	<0.001 *	0.637	−0.01 (−0.01, −0.00)	0.014 *	0.498
1st tertile	Ref	-	0.648	Ref	-	0.501
2nd tertile	−0.19 (−0.53, 0.15)	0.257		−0.01 (−0.01, 0.01)	0.917	
3rd tertile	−1.02 (−1.50, −0.54)	<0.001 *		−0.01 (−0.02, 0.00)	0.068	
PA + Diet						
Did not meet PA recommendation + lower dietary quality	Ref	-	0.652	Ref	-	0.502
Did not meet PA recommendation + higher dietary quality	−0.67 (−1.58, 0.24)	0.145		−0.01 (−0.02, 0.02)	0.868	
Met PA recommendation + lower dietary quality	−1.25 (−1.64, −0.85)	<0.001 *		0.01 (−0.01, 0.01)	0.532	
Met PA recommendation + higher dietary quality	−2.12 (−2.57, −1.68)	<0.001 *		−0.01 (−0.02, 0.00)	0.117	

*Note:* Adjusted β (95% CI), *p*-values and R-square were obtained by performing multiple linear regression (PROC SURVEYREG procedure in SAS), adjusted for sex, race/ethnicity, age, education level, family income to poverty ratio, body mass index and daily energy intake (kcal). NHANES = National Health and Nutrition Examination Survey; PA = physical activity, Ref = the reference group to which other groups compare themselves, HEI = Healthy Eating Index, meeting PA recommendation = 600 MET-minutes physical activity time or more each week; Higher dietary quality = 3rd tertile, 58.1 < HEI ≤ 95.8; lower dietary quality = 1st and 2nd tertiles, 10 ≤ HEI ≤ 58.1. * Symbols indicate statistical significance.

**Table 4:** The sex specific relationship of physical activity and/or dietary quality and android fat composition and distribution, NHANES 2011–2018 (*n* = 10,014).

Variable	Android Percent Fat			Android to Gynoid Ratio		
	Adj. β (95% CI)	*p*-Value	R-Square	Adj. β (95% CI)	*p*-Value	R-Square
**Male**						
PA total-per 100-point increase	−0.0097 (−0.0138, −0.0056)	<0.001 *	0.588	−0.0001 (−0.0002, 0.0000)	0.081	0.326
Insufficiently active	Ref		0.614	Ref		0.334
Active	−0.24 (−0.95, 0.46)	0.49		0.02 (−0.01, 0.05)	0.164	
Highly active	−1.68 (−2.26, −1.09)	<0.001 *		−0.01 (−0.02, 0.01)	0.425	
HEI total-per 10-point increase	−0.29 (−0.52, −0.06)	0.013 *	0.584	−0.01 (−0.01, 0.00)	0.161	0.325
1st tertile	Ref		0.607	Ref		0.332
2nd tertile	−0.83 (−1.35, −0.31)	0.002 *		−0.01 (−0.03, 0.01)	0.433	
3rd tertile	−0.96 (−1.73, −0.19)	0.015 *		−0.01 (−0.03, 0.01)	0.363	
PA + Diet						
Did not meet PA recommendation + lower dietary quality	Ref		0.612	Ref		0.332
Did not meet PA recommendation + higher dietary quality	−0.68 (−2.18, 0.82)	0.371		0.01 (−0.03, 0.05)	0.669	
Met PA recommendation + lower dietary quality	−1.51 (−1.99, −1.03)	<0.001 *		0.01 (−0.02, 0.02)	0.788	
Met PA recommendation + higher dietary quality	−1.98 (−2.74, −1.22)	<0.001 *		−0.01 (−0.03, 0.02)	0.554	
**Female**						
PA total-per 100-point increase	−0.0115 (−0.0169, −0.0060)	<0.001 *	0.59	−0.0001 (−0.0001, 0.0001)	0.91	0.398
Insufficiently active	Ref		0.599	Ref		0.401
Active	−0.57 (−1.29, 0.16)	0.123		0.01 (−0.01, 0.02)	0.55	
Highly active	−1.60 (−2.08, −1.13)	<0.001 *		−0.01 (−0.02, 0.01)	0.579	
HEI total-per 10-point increase	−0.41 (−0.58, −0.25)	<0.001 *	0.591	−0.01 (−0.01, −0.00)	0.003 *	0.4
1st tertile	Ref	-	0.598	Ref	-	0.404
2nd tertile	0.49 (−0.08, 1.07)	0.093		0.01 (−0.01, 0.02)	0.458	
3rd tertile	−1.08 (−1.61, −0.56)	<0.001 *		−0.02 (−0.03, 0.00)	0.026 *	
PA + Diet						
Did not meet PA recommendation + lower dietary quality	Ref		0.602	Ref		0.404
Did not meet PA recommendation + higher dietary quality	−0.76 (−1.60, 0.07)	0.073		−0.01 (−0.03, 0.01)	0.209	
Met PA recommendation + lower dietary quality	−0.96 (−1.53, −0.38)	0.002 *		0.01 (−0.01, 0.02)	0.371	
Met PA recommendation + higher dietary quality	−2.39 (−2.91, −1.87)	<0.001 *		−0.02 (−0.03, −0.00)	0.022 *	
**Interaction term (sex × independent variable)**						
PA total-per 100-point increase	−0.0013 (−0.0071, 0.0046)	0.659	-	−0.0002 (−0.0004, −0.0001)	0.003 *	-
Insufficiently active	Ref		-	Ref		-
Active	0.19 (−0.90, 1.27)	0.733		0.01 (−0.03, 0.04)	0.651	
Highly active	−0.35 (−1.01, 0.30)	0.284		−0.02 (−0.04, 0.00)	0.114	
HEI total-per 10-point increase	0.15 (−0.10, 0.39)	0.231	-	0.01 (−0.00, 0.01)	0.073	-
1st tertile	Ref	-	-	Ref	-	-
2nd tertile	−1.26 (−2.17, −0.35)	0.008 *		−0.01 (−0.03, 0.02)	0.603	
3rd tertile	0.26 (−0.61, 1.12)	0.557		0.02 (−0.00, 0.04)	0.106	
PA + Diet						
Did not meet PA recommendation + lower dietary quality	Ref		-	Ref		-
Did not meet PA recommendation + higher dietary quality	0.10 (−1.48, 1.69)	0.899		0.03 (−0.01, 0.07)	0.172	
Met PA recommendation + lower dietary quality	−0.86 (−1.58, −0.15)	0.019 *		−0.02 (−0.04, 0.01)	0.18	
Met PA recommendation + higher dietary quality	0.21 (−0.68, 1.09)	0.642		0.01 (−0.02, 0.03)	0.592	

*Note:* Adjusted β (95% CI), *p*-values and R-square were obtained by performing multiple linear regression (PROC SURVEYREG procedure in SAS), adjusted for sex, race/ethnicity, age, education level, family income to poverty ratio, body mass index and daily energy intake (kcal). NHANES = National Health and Nutrition Examination Survey; PA = physical activity, Ref = the reference group to which other groups compare themselves, HEI = Healthy Eating Index, meeting PA recommendation = 600 MET-minutes physical activity time or more each week; Higher dietary quality = 3rd tertile, 58.1 < HEI ≤ 95.8; lower dietary quality = 1st and 2nd tertiles, 10 ≤ HEI ≤ 58.1. * Symbols indicate statistical significance.

## Data Availability

Data used for the present study from two publicly accessible datasets: (1) Centers for Disease Control website: https://wwwn.cdc.gov/nchs/nhanes/sasviewer.aspx (accessed on 2 August 2021); and (2) U.S. Department of agriculture website: https://www.ars.usda.gov/northeast-area/beltsville-md-bhnrc/beltsville-human-nutrition-research-center/food-surveys-research-group/docs/fped-data-tables/ (accessed on 2 August 2021).
